# Internalized stigma level, family self-stigma, and family burden of patients receiving community mental health center services: a comparative, longitudinal study

**DOI:** 10.3389/fpsyt.2024.1469448

**Published:** 2024-12-12

**Authors:** Elif Özcan Tozoğlu, Nilifer Gürbüzer

**Affiliations:** Department of Psychiatry, University of Health Sciences, Erzurum Faculty of Medicine, Erzurum, Türkiye

**Keywords:** stigma, schizophrenia, bipolar disorder, community mental health center, self stigma family, caregiver burden

## Abstract

**Introduction:**

The study aimed to evaluate, both comparatively and longitudinally, the effects of receiving services from community mental health centers on the stigma levels of patients and relatives and the burden of care for patients with severe mental illness.

**Methods:**

The study was planned to be conducted on patients with severe mental illness [schizophrenia spectrum disorders (SSDs) and bipolar disorder (BD)] and their relatives, followed by the community mental health center (CMHC group) and the outpatient clinic (outpatient group). It was planned to provide psychoeducation to relatives once a month for 2 h; meetings with the case manager at least once every 2 weeks; and psychosocial interventions (social inclusion, daily life activities studies, etc.) and psychoeducation for 2 h once a week for the patients. The Internalized Stigma of Mental Illness Scale (ISMI) was applied to the patients; the Zarit Caregiver Burden Scale (ZCBS) and the Self-Stigma Inventory for Families (SSI-F) were applied to the relatives at the beginning of the study, at the 6th and 12th month.

**Results:**

The study was completed with 53 patients from the CMHC group (number of patients with SSDs = 39, number of patients with BD = 14) and 60 patients from the outpatient group (number of patients with SSDs = 45, number of patients with BD = 15). In the CMHC group, in patients with SSD, there was a statistically significant decrease in ISMI (*p* < 0.001), ZCBS (*p* < 0.001), and SSI-F (*p* < 0.001) scores at the end of the 12th month. In the outpatient group, in patients with SSD, there was no statistically significant decrease in ISMI (*p* = 0.948), ZCBS (*p* = 1.000), and SSI-F (*p* = 1.000) scores at the end of the 12th month. In the CMHC group, in patients with BD, there was a statistically significant decrease in ISMI (*p* = 0.002), ZCBS (*p* < 0.001), and SSI-F (*p* < 0.001) scores at the end of the 12th month. In the outpatient group, in patients with BD, there was no statistically significant decrease in ISMI (*p* = 0.645), ZCBS (*p* = 0.166), and SSI-F (*p* = 0.142) scores at the end of the 12th month.

**Discussion:**

The results of our study suggest that multidimensional assessments of patients and their families, efforts to promote social participation, support for self-management in daily life, and psychoeducation may be helpful in reducing stigma and burden.

## 1 Introduction

The group most stigmatized in the world are individuals with severe mental illnesses (SMIs) such as schizophrenia spectrum disorders (SSDs) and bipolar disorder (BD), which have a destructive effect on cognitive, managerial, and social skill areas, leading to loss of abilities (1). These individuals, who experience intense stigmatization in almost all areas of their lives, have forms of stigmatization that are almost the same in different countries or regions (1).

In society, having a mental illness is often seen as a sign of personal deficiency, weakness, deviation, low intelligence, unreliability, or incompetence, while it is known that individuals with SMIs are seen as individuals with violent and unpredictable behaviors (2, 3). The stigmatization, which can be briefly defined as the existing false beliefs, negative attitudes, and behaviors towards individuals with mental illnesses, has been shown to be widespread in the general population, including the individuals themselves, their families, their social environments, and among health professionals, according to many study findings (2, 4, 5). Families, in particular, are affected by the stigmatization their patients face and experience feelings of blame, shame, or withdrawal, and concealment. Common beliefs that poor parenting skills trigger mental illnesses and the emergence of the disease as a result of the effect of inheritance biologically cause the stigma on families to be larger and heavier. The perception that they have a shameful feature against society with the thought that the community will interpret the disease of their children as a result of not being able to parent well, not being able to be a good family, outweighs in parents.

The experience of self-stigmatization can lead to a decrease in self-esteem in family members; to despair, helplessness, and depression; to hiding the disease and withdrawing from social relationships; to deterioration in individual and familial functionality; to an increase in burden; and to the disruption of the treatment relationship (6). Determining the burden experienced by the relatives of patients with schizophrenia, the factors associated with the burden, and the initiatives made to reduce the burden is quite important for both the patient individuals and the caregivers.

With the development of social psychiatry, societal treatments have begun to be anticipated as a result of the mindset change in the treatment of SMIs. Changes have been made in mental health policies for the purpose of mental–social treatment. The first step of these changes was the closure of depot hospitals known as mental hospitals and the opening of community mental health centers (CMHCs). Thus, the understanding of the community-based model has begun to dominate the presentation of mental health services instead of the hospital-based model with a medical approach. In this model, which is also recommended by the World Health Organization and is applied more and more as its effectiveness is seen, the country is divided into service regions and each CMHC coordinates and presents mental health services in its own responsible region with a multidisciplinary team consisting of psychiatrists, psychiatric nurses, social workers, psychologists, occupational therapists, and master instructors. CMHCs register mental patients in the responsible region to the center by detecting them and closely monitor the conditions of these patients by also making home and workplace visits when necessary. The understanding of the multidisciplinary team facilitates the provision of multi-faceted services. CMHCs carry out anti-stigma studies with a multidisciplinary approach in the field of mental health and actively support existing studies to increase social functionality and social wellbeing.

According to the report published by the World Health Organization in 2022, the importance of a community-based approach is emphasized in preventing stigmatization (7). It is seen that the initiatives that will reduce stigmatization the most ensure that patients socialize in the same environment as non-patients, guaranteeing that they are employed in the same environment, regaining their abilities to sustain their lives on their own, and even seeing some patients working in jobs beneficial to the community. These initiatives can only be achieved in the current system with individual and multi-faceted studies with a case manager assigned to each patient with CMHCs. One of these studies aims to give psychoeducation to patients and their relatives.

In studies examining the effect of psychoeducation on caregiver burden, it has been shown that psychoeducation reduces the caregiver burden in both schizophrenia and mood disorders (8). In addition, in comparative studies, it has been shown that the quality of life of patients and families who were given psychoeducation increased, their family and social relations improved, and the functionality of the patients increased (9, 10).

There are far fewer studies evaluating the effect of psychoeducation on stigma. In studies conducted with BD, it has been shown that functionality increases and stigma scores decrease in patients given psychoeducation (11). In another study conducted on patients with schizophrenia and schizoaffective disorder, it was shown that stigma scores measured with the social distance scale were lower in the group receiving psychoeducation (12).

The clinical symptoms of schizophrenia and BD often overlap. The BD phenotype includes symptoms such as psychosis, depression, anxiety, sleep disturbances, and cognitive dysfunctions, which cannot always be clearly differentiated from schizophrenia. The “schizophrenia-trait disorder spectrum model” has been put forward to describe this situation. Schizoaffective disorder, in which symptoms related to both diseases coexist, and cases in which the diagnosis changes over time constitute the common points between the two diseases with an average rate of 20% within a spectrum (13). Genetic, neurodevelopmental disorders and biological and psychosocial factors are thought to be important in the etiology of both disorders. Stress and life events may also lead to the emergence of both disorders (13, 14). Schizophrenia and BD have many commonalities in terms of genetic, structural, symptomatic, and therapeutic features. Both disorders are lifelong disorders with a high risk of suicide and prolonged symptomatic periods, and use the health system intensively. Both disorders impose a heavy burden on the individual, family, and society. These illnesses, which occur under the influence of environmental factors and psychosocial stresses, are severe mental disorders whose causes are not fully known and whose treatment can only be partially provided (15). Considering these common factors, both diagnostic groups were addressed in the same study group. At the same time, in terms of stigmatization, in a study conducted in the United Kingdom, many psychiatric diagnoses were evaluated, and it was found that stigmatization was most common in SSDs and most common in BD after personality disorders. It can be said that these two disorders have similar places in the society in terms of stigmatization (16).

We think that CMHCs, which evaluate both the patient and his/her family/relatives more comprehensively, intervene to increase their functionality in many areas, and also give psychoeducation, may have positive effects on stigma on patients and their relatives.

However, when we review the literature, comparative cross-sectional studies have been conducted on the effect of CMHC services on stigmatization before, and no significant positive effect of CMHCs has been detected, and it has been commented that designing the studies as longitudinal studies on the same patients will give more accurate results (17).

In our study, we aimed to evaluate both comparatively and longitudinally the effect of service receipt from the CMHC, the applied psychosocial interventions, and the given psychoeducation on the stigmatization levels of patients with SMI, the stigmatization levels of their relatives, and the burden of care.

## 2 Methods

### 2.1 Ethics and consent

The research protocol was approved by the Scientific Research Ethics Committee of Health Sciences University Erzurum Faculty of Medicine (Erzurum, Türkiye) with the decision numbered BAEK 2023/01-01 and carried out in accordance with the Helsinki Declaration. Written informed consent was obtained, stating that they agreed to participate in the study and gave their consent to the publication of all clinical and other data contained in the manuscript from all participants and/or their court-appointed guardians, if any.

### 2.2 Participants

The study was planned to be conducted on patients with SMI who are followed by the community mental health center (CMHC group) and the outpatient clinic (outpatient group) between June 2023 and May 2024. The inclusion criteria were as follows: being between the ages of 18 and 60; having a diagnosis of SSDs and BD made by a psychiatrist by applying SCID-5; the patient being in remission during the study process [patients with SSDs are in the remission phase according to Van os criteria (18); patients with BD with a Young Mania Rating Scale ≤8 and a Hamilton Depression Scale ≤7 for the last 6 months (19)]; the patient and the relative having the literacy skills to understand and fill out the tests; the patient and, if any, his guardian or a relative agrees to participate in the study; and showing that the patient’s relative does not have a mental disorder through the application of SCID-5 by a psychiatrist. The exclusion criteria were as follows: the patient and the relative having mental capacity problems, having an oncological pathology that can increase the care burden in the patient or the relative, and not being able to maintain more than 20% of the trainings.

### 2.3 Study design and setting

The Erzurum Community Mental Health Center is currently monitoring 908 patients (681 with SSDs and 227 with BD) with severe mental disorders. The center’s staff (doctors, psychologists, occupational therapist, nurses, social worker, and occupational technician) have received online training, on-the-job training, and international supervision training as part of the CMHC trainings jointly organized by the Ministry of Health and the World Health Organization. An occupational therapist, an occupational technician, and a psychologist who received the training had been working in the field of mental health for at least 4 years; nurses and doctors had been working in the field of mental health for at least 10 years. Their competencies related to the training provided were evaluated. Personnel and the work carried out were inspected twice a year by the Ministry of Health and the Provincial Health Directorate Mental Health Department.

Case management is being done for registered patients, their pharmacological treatments are monitored by the doctor, and field studies aimed at improving abilities impaired by the effects of the disease, especially negative symptoms, family trainings, and home/workplace visits, are being made.

For patients who met the work criteria and agreed to participate in the study and their primary caregiver, in addition to the individual psychosocial interventions of the case manager specific to the patient’s needs, psychoeducation was planned for 1 year.

The case manager got to know the patient, identified their strengths, and provided interactive guidance. Support was provided for further education and job interviews. Course guidance was provided, and study plans were organized to prepare for exams for people with disabilities.

The case manager aimed to increase family functionality by discussing duties and responsibilities within the family. Discussions were held to improve friend relationships, if any. Alternative ways to make friends were discussed. They were guided and supported to participate in handicraft courses, job training courses, and cultural clubs. On these and similar issues, the case manager worked individually to meet the needs of the patient.

Psychoeducation lasting 2 h each month was planned for patient relatives/caregivers; at least once every 2 weeks, individual face-to-face meetings with the case manager and psychoeducation lasting 2 h once a week were planned for patients. Care4Today materials were used for psychoeducation.

Before starting the study, patients and their relatives were informed about the study, and their written and verbal consents were obtained. At the beginning of the study, the sociodemographic data form was applied to the patients and the Internalized Stigma of Mental Illness Scale (ISMI), the Zarit Caregiver Burden Scale (ZCBS), and the Self-Stigma Inventory for Families (SSI-F) were applied at the beginning of the study, at the 6th month of follow-up, and at the 12th month. If the patient’s relative does not meet the criteria but the patient does, the patient was included in the training but was not included in the study data.

To prevent bias, during the process, the scales were filled out by a single psychiatrist who was not involved in the patients’ psychoeducation and psychosocial interventions and did not perform case management for any patient.

### 2.4 Psychosocial interventions

A care plan is created for each patient’s individual needs. In this plan, the goal is reached in line with the aim and with the strategies determined.

#### 2.4.1 Studies in the field of occupational activities and educational needs

A care plan was created for the occupational and educational needs of the patients. In this context, strategies to overcome the obstacles in meeting these needs with the work were carried out; returning to work or school, skill development, and social interaction studies were included in the plan.

In the community, advocacy work was carried out on behalf of service recipients to combat discrimination and stigma that existed and pose an obstacle to reaching the goals of education and occupational therapy.

Advocacy was defined as working with or on behalf of service recipients to contribute to the change of policies or practices that have a negative impact on service recipients or to access services or resources that could not be obtained without effort.

#### 2.4.2 Studies aimed at improving daily life activities

It is ideal that patients meet their daily life needs independently. Daily life activities were included in the care plan. In this context, strong, weak, and vulnerable aspects of service recipients and strategies to cope with them were also included in the plan. Practical support was provided to service recipients to meet their basic needs, if necessary, in cooperation with other community support institutions and caregivers.

#### 2.4.3 Social inclusion studies

Social inclusion means that individuals fully and actively participate in society, where they are valued and respected despite their differences, where their basic needs are met, and where they live with human dignity. Work is done together with service recipients to enable them to participate in all aspects of community life and to be able to contribute. In the care plan, studies are included to increase social inclusion and to address the obstacles to inclusion. In this context, strategies to reduce isolation and marginalization, strengthen the ties of service recipients with the community, and increase their self-confidence are also included in the plan.

#### 2.4.4 Psychoeducation

Care4 Today Healthy Living Guide was used as material for psychoeducation (20). Care4Today is a psychoeducation program developed for patients with schizophrenia and their families to be used under the guidance of trained practitioners. This psychoeducation program mainly includes symptoms of schizophrenia, diagnosis of schizophrenia, causes of schizophrenia, effects and side effects of medications, exacerbation symptoms, crisis planning, disease management and recovery, and coping with drugs and alcohol. In addition to psychoeducation, it is aimed that patients lead healthier lives with modules on healthy nutrition and shopping, physical activity and exercise, quitting smoking, communication, relationships, and sexuality. It has been shown that the program increases patients’ adherence to treatment and reduces hospital stay time and treatment costs (21).

The Healthy Living Guide includes Workbooks Facilitator Guide, Healthy Living Activities Implementation Guide, Patient Workbooks, Healthy Living Books, and visual materials in presentation and video format.

In the presentations, owing to the lack of information specific to BD, additional presentations on the symptoms, diagnosis, and causes of BD were prepared by our team and included in the psychoeducation.

### 2.5 Data sources/measurement

#### 2.5.1 Sociodemographic information form

This form is designed to evaluate the sociodemographic information (such as age, sex, and working status) and clinical characteristics (such as total disease duration and total number of hospitalizations) of the participants in the study.

#### 2.5.2 Structured Clinical Interview for DSM-5 Disorders, clinician version

The Structured Clinical Interview for DSM (SCID) is one of the most widely used diagnostic tools in clinical research worldwide. The latest version is SCID-5. SCID-5, Clinician Version is a comprehensive, standardized tool for the evaluation of major psychiatric disorders according to the definitions and criteria of DSM-5. This structured clinical interview includes 32 diagnostic categories with detailed diagnostic criteria and 17 diagnostic categories with research questions. The validity and reliability study of the Turkish version of SCID-5, Clinician Version was conducted (22).

#### 2.5.3 Hamilton Depression Scale

It assesses the severity of depressive symptoms over 17 items and on a three- or five-level dimension. It is filled out by the clinician, and the total score indicates the severity of depression. A score between 0 and 7 indicates absence of depression (23). In the Turkish adaptation study, the Cronbach’s alpha value was found to be 0.75 (24).

#### 2.5.4 Young Mania Rating Scale

It measures core symptoms defined for the manic period of BD, each one through 11 items with five stages (from mild to severe). The scale is filled out based on an interview taking into account the patient’s condition in the last 48 h (25). In the Turkish validity and reliability study of the scale, the internal consistency coefficient was found to be 0.79 (26).

#### 2.5.5 Internalized Stigma of Mental Illness Scale

The ISMI was developed in 2003 by Ritsher and colleagues, and the validity and reliability study of the Turkish version was conducted by Ersoy and Varan (2007). This self-report scale, which evaluates internalized stigma in psychiatric patients, consists of a total of 29 items. The scale has five subdimensions: alienation, endorsement of stereotypes, perceived discrimination, social withdrawal, and resistance to stigma. In the four-point Likert-type scale, the items of the resistance to stigma subscale are scored inversely, and an increase in the scale score indicates that the person’s level of internalized stigma is high in a negative direction. While the Cronbach’s alpha coefficients for the subscales of ISMI vary between 0.63 and 0.87, the Cronbach’s alpha coefficient for the entire scale is calculated as 0.93 (27).

#### 2.5.6 Zarit Caregiver Burden Scale

The ZCBS was developed by Zarit and colleagues in 1980. It is a scale used to evaluate the difficulty experienced by caregivers providing care to an individual in need of care. It can be filled out by the caregiver themselves or by the researcher. The items on the scale are generally oriented towards the social and emotional area, and a high score on the scale indicates that the experienced difficulty is high. The validity and reliability study of the scale for the Turkish version has been conducted (28). The Cronbach’s alpha coefficient of the scale was found to be 0.95 (29).

#### 2.5.7 Self-Stigma Inventory for Families

The Self-Stigma Inventory for Families (SSI-F) is a scale consisting of 14 items and three factors, withdrawal from society, hiding the disease, and perception of worthlessness, aimed at evaluating self-stigmatization or internalized stigmatization in individuals with mental illness. The five-point Likert-type measurement was designed as follows: “1 = does not fit me at all, 2 = fits a little, 3 = fits moderately, 4 = usually fits, 5 = fits perfectly”. As the scores obtained from the scale increase, the self-stigmatization of the patient’s relatives increases. The Cronbach’s alpha coefficient of the scale was found to be 0.88 (30).

### 2.6 Statistical analysis

Analyses were conducted using the IBM SPSS 26 statistical analysis software. The data were presented in terms of mean, standard deviation, and count. A normality analysis was performed to check whether the skewness and kurtosis values of all variables fall within the range of −2 to +2. These values indicate that the normality assumption is met (George, 2011). Since the normal distribution condition was met, the independent samples test was used for comparisons between two independent groups. The chi-square test was applied for comparisons between categorical variables.

To evaluate the change in scales over time, a repeated-measures analysis of variance (ANOVA) was performed. Partial eta squared (*η*_partial_^2^) values were given to determine the effect size. The assumption of sphericity was evaluated with Mauchly’s Sphericity test. In cases where the data met the assumption of sphericity, the “Sphericity” test was used; in cases where this assumption was not met, the “Greenhouse Geisser” test was used. Bonferroni multiple comparison tests were used to analyze the mean differences between the groups.

A repeated-measures ANOVA test assessed intra-group comparison (overall) analyses across multiple time points. Bonferroni multiple comparison tests were used to analyze the mean differences between the groups.

Pearson correlation analysis was performed to evaluate the correlation between scale scores. A linear regression analysis was performed to investigate the effects of common variables in a mixed model containing scale scores. The level of statistical significance was taken as *p* < 0.05.

## 3 Results

The study started with 65 CMHC patients and an equal number of 65 outpatient groups who met the inclusion and exclusion criteria. However, because of the acute phase of one patient with SSDs during the process, one BD patient being hospitalized and treated due to a manic attack, seven patients and three patient relatives not attending more than 20% of psychoeducations, their data were not included in the study. From the outpatient group, two patients with BD were removed from the study due to manic attack, one BD patient was removed due to depressive attack, and two patients with SSDs entering the acute phase and who were hospitalized were removed.

The study was completed with 53 patients from the CMHC group (number of SSDs = 39, number of BDs = 14) and 60 patients from the outpatient group (number of SSDs = 45, number of BDs = 15). The sociodemographic data of the groups are shown in **Table 1**. Skewness-kurtosis analysis was performed to evaluate whether the data fit the normal distribution. It was seen that the data fit the normal distribution.

**Table 1:** Comparison of socio-demographic data and first examination scores of the groups.

		CMHC group *N*/mean ± SD	Outpatient group *N*/mean ± SD	*χ* ^2^/*t*	*p*-value
Age of patient		39.89 ± 9.62	42.60 ± 8.80	−1.566	0.12
Disease duration (years)		12.77 ± 6.25	13.47 ± 5.98	−0.602	0.548
Number of hospitalizations		3.68 ± 5.16	3.82 ± 2.90	−0.177	0.86
Education time of patient (years)		9.37 ± 3.40	7.40 ± 3.34	3.117	0.002
Age of patient relative		50.11 ± 9.79	49.62 ± 10.93	0.253	0.801
Education time of patient relative		7.51 ± 3.43	7.50 ± 3.56	0.014	0.989
ZCBS_First Examination_		46.23 ± 21.74	52.88 ± 22.00	−1.614	0.109
SSI-F_First Examination_		33.08 ± 13.56	22.33 ± 12.28	4.419	0
ISMI_First Examination_		77.36 ± 15.16	70.30 ± 19.10	2.156	0.033
Sex of patient	Men	38	38	0.894	0.344
	Women	15	22		
Marital status of patient	Maried	18	21	0.013	0.908
	Single	35	39		
Working status of patient	Not working	21	37	9.024	0.108
	Works iregularly	11	9		
	Works regularly	14	10		
	Student	2	0		
	Retired	2	0		
	Housewife	3	4		
Sex of patient relative	Men	28	27	0.691	0.406
	Women	35	33		
Working status of patient relative	Not working	6	4	12.773	0.012
	Works iregularly	2	0		
	Works regularly	17	7		
	Retired	11	14		
	Housewife	17	35		
Status of closeness of the patient relative	Partner	16	12	15.552	0.004
	Parents	24	34		
	Child	1	9		
	Second-degree relatives	1	3		
	Sibling	11	2		

**Figure 1:**
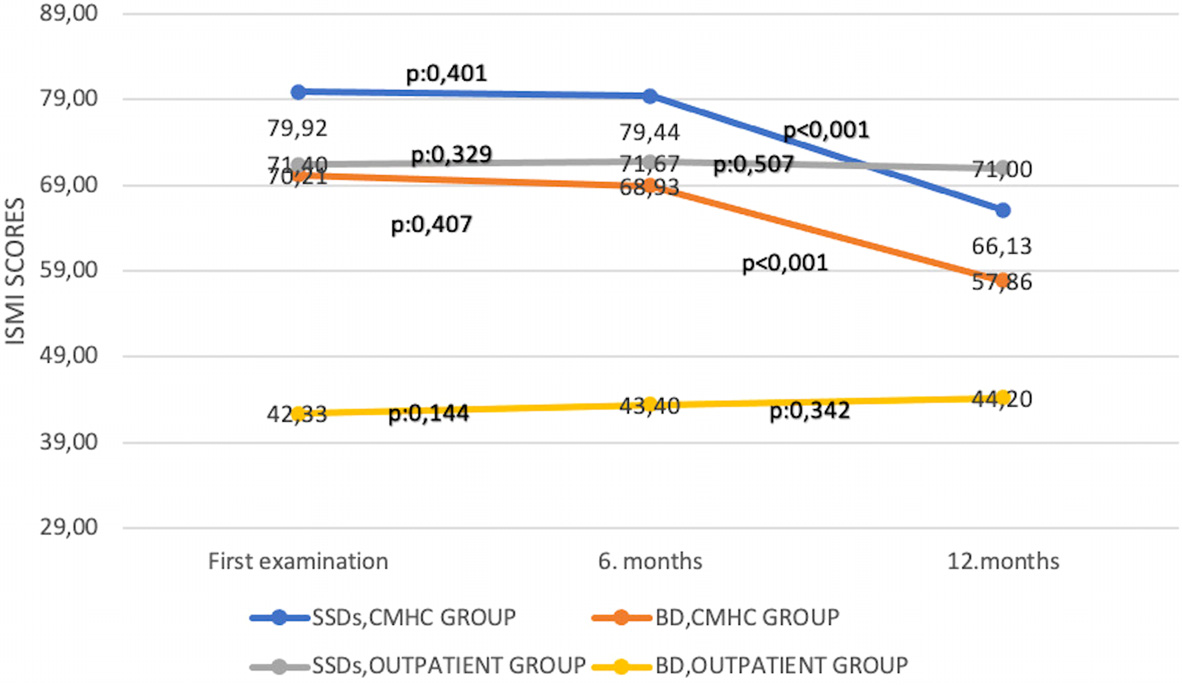
Change of Internalized Stigma of Mental Illness Scale scores over time. BD, bipolar disorder; CMHC, community mental health center; SSDs, schizophrenia spectrum disorders; ISMI, Internalized Stigma of Mental Illness Scale.

**Figure 2:**
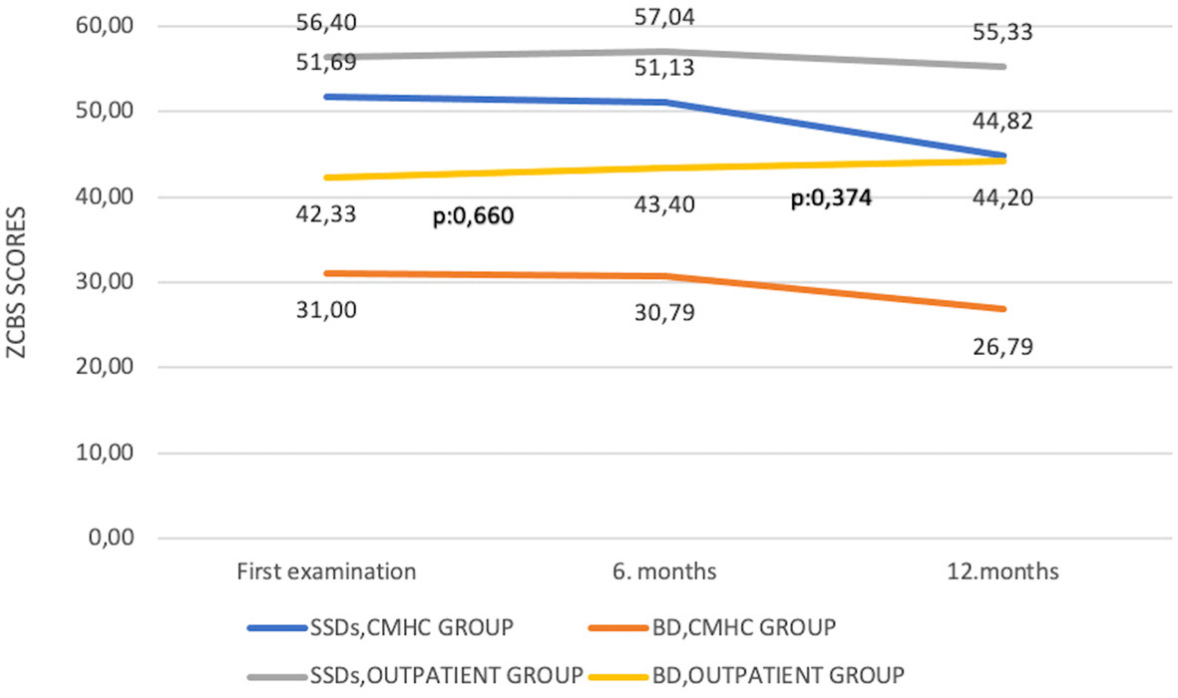
Change of Zarit Caregiver Burden Scale scores over time. BD, bipolar disorder; CMHC, community mental health center; SSDs, schizophrenia spectrum disorders; ZCBS, Zarit Caregiver Burden Scale.

**Figure 3:**
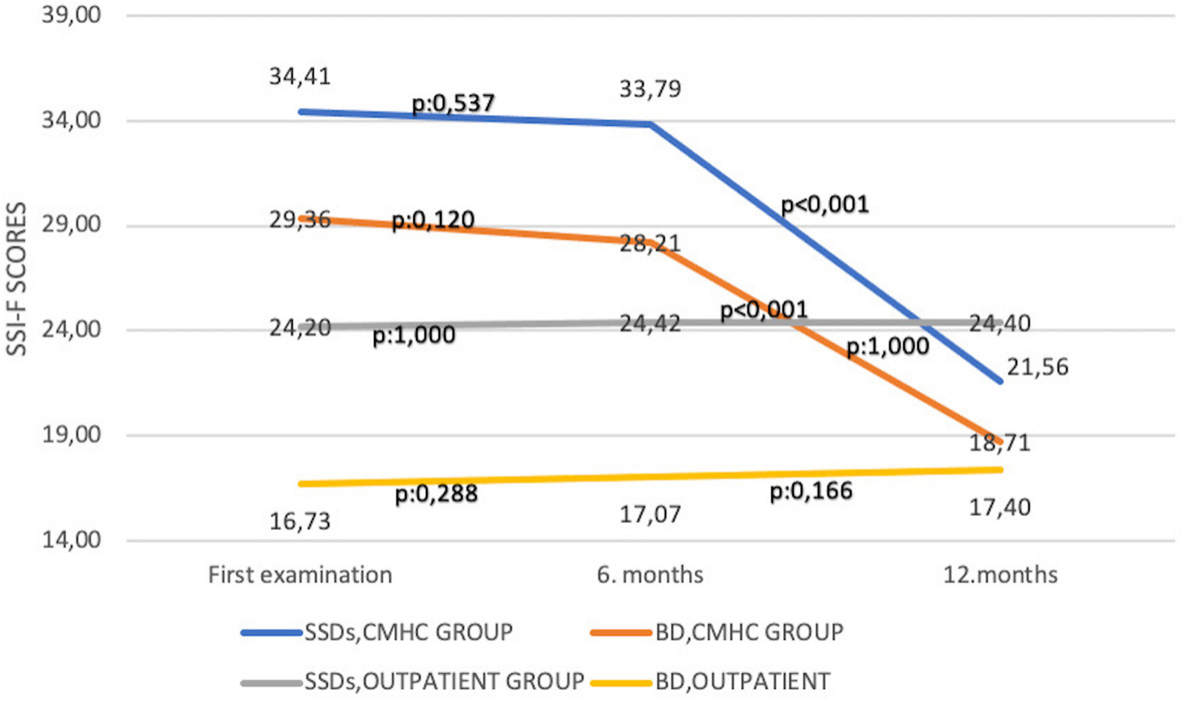
Change of Self-Stigma Inventory for Families Scale scores over time. BD, bipolar disorder; CMHC, community mental health center; SSDs, schizophrenia spectrum disorders; SSI-F, Self-Stigma Inventory for Families.

The initial scores of the CMHC and outpatient group were evaluated with the Student *t*-test (**Table 1**). To evaluate the change of ISMI, ZCBS, and SSI-F scores over time, ANOVA test was applied separately to each group. In the CMHC group, in patients with SSDs, there was a statistically significant decrease in ISMI (*F* = 214,972, *η*_partial_^2^ = 0.850. *p* < 0.001), ZCBS (*F* = 38,084, *η*_partial_^2^ = 0.501, *p* < 0.001), and SSI-F (*F* = 123,523, *η*_partial_^2^ = 0.765, *p* < 0.001) scores at the end of the 12th month. In the results of the Bonferroni corrected analysis performed to determine from which time the difference found originated, it was seen that the difference between the first interview and the 12th month and between the 6th and 12th month was statistically significant. In the outpatient group, in patients with SSDs, there was no statistically significant decrease in ISMI (*F* = 1,371, *η*_partial_^2^ = 0.030. *p* = 0.948), ZCBS (*F* = 1,702, *η*_partial_^2^ = 0.037, *p* = 1.000), and SSI-F (*F* = 0.235, *η*_partial_^2^ = 0.005, *p* = 1.000) scores at the end of the 12th month. The change of ISMI, ZCBS, and SSI-F scores over time in both groups and the *p*-values applied with Bonferroni correction are shown in **Figures 1**–**3**.

In the CMHC group, in patients with BD, there was a statistically significant decrease in ISMI (*F* = 59,505, *η*_partial_^2^ = 0.821, *p* = 0.002), ZCBS (*F* = 21,918, *η*_partial_^2^ = 0.850. *p* < 0.001), and SSI-F (*F* = 28,361, *η*_partial_^2^ = 0.686, *p* < 0.001) scores at the end of the 12th month. In the results of the Bonferroni-corrected analysis performed to determine from which time the difference found originated, it was seen that the difference between the first interview and the 12th month and between the 6th and 12th month was statistically significant. In the outpatient group, in patients with BD, there was no statistically significant decrease in ISMI (*F* = 3,890, *η*_partial_^2^ = 0.217, *p* = 0.645), ZCBS (*F* = 1,099, *η*_partial_^2^ = 0.073, *p* = 0.166), and SSI-F (*F* = 3,182, *η*_partial_^2^ = 0.185, *p* = 0.142) scores at the end of the 12th month. The change of ISMI, ZCBS, and SSI-F scores over time in both groups and the *p*-values applied with Bonferroni correction are shown in **Figures 1**–**3**. In the groups where ISMI scores were significant, subscales were evaluated. The change of ISMI subscales over time in patients with SSDs and BD in the CMHC group is shown in **Table 2**. In the BD group, except for the resistance to stigma subscale scores, statistically significant decreases were detected in all scores at the end of the 12th month.

**Table 2:** Change of Internalized Stigma of Mental Illness subscale scores over time.

ISMI Subscales			Mean ± SD	*p*-value with Bonferroni correction	*p*-value
SSDs	Stigma resistance	First examination	14.59	0.971 *p* < 0.001	*p* < 0.001
		6.ay	14.56		
		12.ay	13.26		
	Alienation	First examination	15.74	0.69 *p* < 0.001	*p* < 0.001
		6.ay	15.62		
		12.ay	12.95		
	Stereotype endorsement	First examination	17.26	0.48 *p* < 0.001	*p* < 0.001
		6.ay	17.31		
		12.ay	15.46		
	Social withdrawal	First examination	17.85	0.448 *p* < 0.001	*p* < 0.001
		6.ay	17.46		
		12.ay	13.49		
	Perceived discrimination	First examination	14.49	1 *p* < 0.001	*p* < 0.001
		6.ay	14.49		
		12.ay	10.97		
BD	Stigma resistance	First examination	14.5	1 0.23	0.689
		6.ay	14.71		
		12.ay	13.93		
	Alienation	First examination	13.5	1 0.015	0.016
		6.ay	13		
		12.ay	10.36		
	Stereotype endorsement	First examination	14.79	0.494 0.013	0.007
		6.ay	14.64		
		12.ay	13.21		
	Social withdrawal	First examination	15.57	0.87 0.009	*p* < 0.001
		6.ay	14.79		
		12.ay	11.64		
	Perceived discrimination	First examination	11.86	1 *p* < 0.001	*p* < 0.001
		6.ay	11.79		
		12.ay	8.71		

The change of SSI-F subscales over time in patients with SSDs and BD in the CMHC group is shown in **Table 3**. In both groups, statistically significant decreases were detected in SSI-F subscales at the end of the 12th month.

**Table 3:** Change of Self-Stigma Inventory for Families subscale scores over time.

ISMI Subscales			Mean ± SD	*p*-value with Bonferroni correction	*p*-value
SSDs	Social withdrawal	First examination	14.67 ± 5.70	1 *p* < 0.001	*p* < 0.001
		6th month	14.51 ± 5.48		
		12th month	8.82 ± 3.56		
	Concealment of the illness	First examination	7.56 ± 0.43	0.269 *p* < 0.001	*p* < 0.001
		6th month	7.38 ± 0.42		
		12th month	4.56 ± 0.31		
	Perceived devaluation	First examination	12.46 ± 0.75	0.579 *p* < 0.001	*p* < 0.001
		6th month	12.26 ± 0.71		
		12th month	8.13 ± 0.63		
BD	Social withdrawal	First examination	12.50 ± 6.24	0.204 *p* < 0.001	*p* < 0.001
		6th month	12.00 ± 6.00		
		12th month	7.93 ± 4.09		
	Concealment of the illness	First examination	6.50 ± 2.98	0.568 *p* < 0.001	*p* < 0.001
		6th month	6.29 ± 2.95		
		12th month	4.00 ± 1.84		
	Perceived devaluation	First examination	10.79 ± 5.15	0.086 *p* < 0.001	*p* < 0.001
		6th month	10.29 ± 5.11		
		12th month	6.71 ± 3.29		

The correlation of scale scores at the end of the 12th month with each other was examined. A moderate level of significant and positive relationship was found between ZCBS and SSI-F total score and subscale scores. SSI-F total scale score, ISMI total score, and social withdrawal and perceived discrimination subscale scores were moderately significant and positively related. ISMI total score and alienation, confirmation of stereotypes, social withdrawal, and perceived discrimination subscale scores showed a moderate level of significant and positive relationship with ZCBS scores (**Table 4**).

**Table 4:** Correlation analysis of scale and subscale scores of community mental health center patients at 12 months.

		1	2	2a	2b	2c	3	3a	3b	3c	3d	3e
1. ZCBS	*r*	-	0.341	0.312	0.354	0.344	0.449	−0.122	0.401	0.395	0.37	0.363
	*p*	-	0.012	0.023	0.009	0.012	0.001	0.383	0.003	0.003	0.006	0.007
2. SSF-I	*r*			0.977	0.993	0.978	0.327	−0.266	0.211	0.251	0.311	0.559
	*p*			0.000	0.000	0.000	0.017	0.054	0.13	0.07	0.024	0.000
2a. Social withdrawal	*r*				0.971	0.915	0.367	−0.253	0.23	0.284	0.352	0.585
	*p*				0.000	0.000	0.007	0.067	0.097	0.039	0.01	0.000
2b. Concealment of the illness	*r*					0.969	0.337	−0.225	0.208	0.267	0.314	0.537
	*p*					0.000	0.014	0.106	0.136	0.053	0.022	0.000
2c. Perceived devaluation	*r*						0.286	−0.26	0.18	0.209	0.285	0.523
	*p*						0.038	0.06	0.197	0.133	0.038	0.000
3. ISMI	*r*							−0.023	0.879	0.748	0.802	0.773
	*p*							0.871	0.000	0.000	0.000	0.000
3a. Stigma resistance	*r*								−0.078	−0.44	−0.172	−0.234
	*p*								0.577	0.001	0.218	0.091
3b. Alienation	*r*									0.572	0.653	0.583
	*p*									0.000	0.000	0.000
3c. Stereotype endorsement	*r*										0.533	0.652
	*p*										0.000	0.000
3d. Social withdrawal	*r*											0.506
	*p*											0.000
3e. Perceived discrimination	*r*											-
	*p*											-

A linear regression analysis was performed to investigate the effects of common variables in a mixed model containing ZCBS, ISMI, and SSI-F 12th month total scale scores. According to the analysis result, it was found that ISMI total score was effective for ZCBS (B ± SE; 0.509 ± 0.179, *p* = 0.006), and in the model created for ISMI (B ± SE; 0.283 ± 0.098, *p* = 0.006), ZCBS total score was effective (**Table 5**).

**Table 5:** Linear regression analysis of 12th-month scale scores of community mental health center patients.

Parameters	Independent variables	*B* (95%Cl)	OR	*p*-value	*R* ^2^/adjusted *R* ^2^	*p*-value for *F* change
ZCBS	Model				0.244/0.213	0.001
	SSF-I	0.414 (−0.083/0.912)	0.218	0.101		
	ISMI	0.509 (0.157/0.862)	0.378	0.006		
SSI-F	Model				0.154/0.120	0.015
	ZCBS	0.128 (−0.026/0.281)	0.243	0.101		
	ISMI	0.154 (−0.053/0.361)	0.213	0.141		
ISMI	Model				0.235/0.205	0.001
	ZCBS	0.283 (0.087/0.479)	0.382	0.006		
	SSF-I	0.277 (−0.095/0.650)	0.197	0.141		

## 4 Discussion

Stigmatization related to SMI is a universal problem and is prevalent in every culture and everywhere. People living with mental health conditions can experience stigmatization from their families, neighbors, and health professionals (31). In addition to this, there is also the stigma internalized by the patients. Because people generally prefer to endure mental distress without relief, there is a risk of discrimination and exclusion that comes with access to mental health services. Although the symptoms of the disease can be reduced with pharmacotherapy, negative symptoms that are difficult to control, perhaps the care burden it brings, withdrawal from the social environment, and stigmatization cannot be solved with pharmacotherapy. However, with the right support, most people with serious mental health conditions can maintain excellent relationships and be functional in many areas. Therefore, addressing stigmatization in studies is an important responsibility. The role of CMHCs, which carry out community-based recovery that came to the agenda with social psychiatry, is important in this regard. In our study, when SMI patients followed in the outpatient clinic were compared with SMI patients followed in the CMHC; although the initial scale scores were similar; although not in the short term, in the long term, with the interventions applied in the CMHC, it was shown that the levels of stigma in themselves and their families were reduced in many areas, and the caregiver burden in their caregivers was significantly reduced.

While the symptoms of stigmatization can sometimes be described as worse than the symptoms of their illness by patients with SMI, when the literature is reviewed, it is seen that studies aimed at reducing stigmatization and discrimination are generally conducted by high-income countries (32–34). Studies conducted in low- and middle-income countries are much less. In a study conducted in China in 2018, a community-based intervention was discussed. In the randomized, controlled, and 9-month longitudinal study, despite the joint implementation of the program-named strategies against discrimination and stigmatization in addition to psychoeducation, social skills training, and cognitive-behavioral therapy, it was shown that there was no significant decrease in the ISMI scores in the intervention group, but there were significant improvements in the scores of anticipated discrimination and overcoming stigma compared to the control group. Only patients with schizophrenia were included in this study, and no studies were conducted on patients with BD. A study of community-based interventions with 30 patients with schizophrenia and their families was conducted in India. In the study, a model was created that included psychoeducation (providing information about the disease), adherence management (increasing regular and correct use of medications through adherence strategies and side effect management), rehabilitation (improving functional abilities through social, vocational, and other skills training and planning of daily activities), and referral to community organizations (increasing community support by increasing knowledge and access to disability benefits, employment agencies, and social welfare organizations), as well as self-care practices, appropriate diet and lifestyle changes, and stress and anger management. Although many difficulties were encountered during the implementation of the model, such as not reaching the targeted number of sessions and experiences of stigmatization and discrimination not being adequately addressed, the community-based approach in schizophrenia has been accepted as an acceptable and feasible approach, especially to reduce the treatment gap (35). Again, in India, four people with SMI from a family were included in a community-based intervention program. The main elements of the program were home visits, one-to-one interaction, collaborative work with local government bodies, medical intervention, social work team interventions, social skills training and vocational training, and psychoeducation including information-education and communication skills. After the program, family burden and stigma scores of patients with SMI decreased compared to before the program. The results of the study were similar to those of our study. It has been interpreted that community-based intervention can provide changes in stigmatization, reduce discrimination, and increase social acceptance and social support of family members (36). A yearlong multicenter study similar to our study was conducted in India to evaluate the effectiveness of a community-based intervention for patients with schizophrenia and caregivers. While 167 patients with schizophrenia and their families received community-based intervention, 86 patients and their families received only hospital care. However, unlike our study, stigmatization was not evaluated in this study; disease severity and disability were evaluated. At the end of 1 year, there were significant decreases in the scores of the positive and negative syndrome scale and disability assessment scale in the community-based intervention group compared to the hospital group (37). In a study published in 2021, a 30-year evaluation of interventions to reduce mental health stigma in India was conducted. Only nine studies published on stigmatization and including pharmacotherapy interventions could be included in the review. Only one of these studies dealt with patients with schizophrenia and their relatives. In this review, it was suggested that social contact was the most effective strategy to reduce mental health stigma. However, it was also interesting that there was only one study in the review that used social contact as part of the intervention (38). This review also shows that there are very few studies on stigmatization and community-based interventions, especially in underdeveloped and developing countries. When studies conducted in our country are examined, it is seen that most of the studies conducted in the field of stigma towards severe mental disorders are descriptive (5, 39, 40), and in the studies where interventions are made, the intervention consists of psychoeducation (11, 41); it is seen that there is no study that integrates community-based interventions into the health system and measures its effect longitudinally.

In our study, although not specific to stigmatization, a psychoeducation program was implemented that included interactive information sharing about identifying the disease, informing about its treatment, and discussions on what can be done to reduce flare-ups and increase functionality. In addition, each patient in the CMHC group has a case manager. The case manager first gets to know the patient well in all aspects. It identifies the patient’s strengths and guides the patient through interactive conversations to be able to do again what they could do with these strengths before the disease. It is shown that the patient can contribute to himself and life by doing what he can do not in the CMHC environment but in the community. If he has an interrupted education process, he is supported to continue. If necessary, a rehearsal is made before the job interviews that need to be done for employment purposes, and if necessary, the case manager goes to the job interview with him. Again, for employment purposes, it directs the patient to courses in preparation for public personnel exams for the disabled and organizes exam study plans. If the patient is working at a workplace, a workplace visit is made and the patient is visited while there. It is discussed what can be done at the place where he works. What can be done to make friends according to the patient’s location is also discussed. While these are being discussed, patients and their relatives are confronted with the fact that a person with any physical illness or a person without an illness also struggles with these issues, and while doing this, it is tried to discuss the stigmatization internalized by the patient. Seeing that the patient is in the community also reduces the stigma and burden brought by the disease for the patient's relatives.

As seen in our study, patients and their relatives establish a connection with the case manager, seeing that the recommendations work, and the improvement of the feeling of exclusion due to the disease is a situation that requires time. It has been thought that positive results may not have been observed because the studies showing the effectiveness of CMHCs on the subject of stigmatization are generally cross-sectional comparative studies (17). However, as in our study, when long-term individual and group studies are conducted on the same patient and relative and worked with a community-based logic, a decrease in stigmatization levels in the family and patient and a decrease in caregiver burden have been observed.

Charlene Sunkel, the founder of the Global Mental Health Peer Network and co-chair of the Lancet Commission on Ending Stigma and Discrimination in Mental Health, says, “If there’s one solution to resolving stigma, it’s inclusion of people with mental health conditions in everything—in employment, education, communities. By including people, others can see it’s another human being, deserving of dignity and human rights” (42). In our study, not only did we support patients in psychoeducation, we also strived for each patient in the CMHC group to be included in society as if they were an individual without a mental illness. The results of our study show that the individual and multifaceted evaluation of patients and their relatives, working for social inclusion, enabling the patient to manage themselves in daily life, and community-based interventions are effective ways to reduce stigma and burden.

The effects of stigma on individuals with SMI include perceived, experienced, anticipated, and self-stigma. Perceived stigma is defined as an individual's beliefs about the general public's attitudes toward individuals with SMI. A study of 422 patients with SMI from a psychiatric hospital and four CMHCs in China found that perceived public stigma was a primary condition for individual stigma to occur (43). Experienced stigma refers to the discrimination experienced by people with SMI. A study conducted in the United States on 516 patients with SMI showed that experienced stigma, but not self-esteem and self-efficacy, was a predictive factor for internalized stigma (44). Anticipated stigma—or the expectation that a person will be discriminated against because of their SMI—can occur even if the person has no prior experience of discrimination and contributes to social withdrawal and self-stigma. Self-stigma—or internalized stigma—describes the transformation process in which a person’s previous social identity (defined by social roles such as son, sibling, friend, employee, or potential spouse) is increasingly replaced by a devalued and stigmatized view of the self, referred to as the “disease identity.” In our study, we focused on the internalized stigma that the patient has. However, other effects of stigma are also important and should not be ignored. Future studies that include more comprehensive studies would be valuable. Family stigma is a stigma that arises from others' negative perceptions, attitudes, feelings, and avoidant behaviors towards a family (and each family member); from others' beliefs that the family's unusualness is somehow harmful, dangerous, and unhealthy, which may affect them negatively or is different; and from the family's social isolation due to the illness identity of their patients. In our study, we evaluated the families' own stigmatization, but it may be instructive to show all these effects on family stigmatization in more comprehensive studies.

Although there are many things that can be done publicly and as mental health professionals in order to reduce stigmatization, we were able to examine only one pillar of psychoeducation and psychosocial interventions in our study (45). The most significant limitation of the study is that a structured program aimed at combating self-stigmatization was not implemented in the study. As there are studies showing the positive effect of psychosocial struggle programs used against self-stigmatization in SSDs in psychoeducation (46), there are also studies showing that programs specifically applied to stigmatization have no effect (47). Another limitation is that the Care4Today application is a program prepared only for patients with schizophrenia. However, the fact that schizophrenia and BD have many similar aspects both etiologically and clinically, and that we as a team prepare BD-specific materials and presentations for the program, partially reduces this limitation.

The longitudinal design of the study with the same patients constitutes a strong aspect of the study. However, there is clearly a need for studies involving longer-term follow-up to assess whether the initial gains are maintained or reduced and whether booster doses of intervention are needed to sustain progress. Making individual plans for each of the patients and their relatives, conducting multidimensional evaluations (from the perspectives of occupational therapists, psychologists, and doctors), and including the family in the study are among the strengths of the study. While most studies on community-based interventions and stigmatization included only patients with schizophrenia, the inclusion of patients with BD in our study makes it stand out. The bias was eliminated by having the scales filled out by a single psychiatrist who was not involved in the psychoeducation and psychosocial interventions of the patients and who did not do case management for any patients throughout the process. However, considering the limitations we mentioned, conducting multicenter studies in the future, including country policies and systematic cooperation with institutions, may reveal the effect of community-based interventions on stigma more clearly.

## Data Availability

The raw data supporting the conclusions of this article will be made available by the authors, without undue reservation.
